# Effect of scutellarin on BV-2 microglial-mediated apoptosis in PC12 cells via JAK2/STAT3 signalling pathway

**DOI:** 10.1038/s41598-024-64226-x

**Published:** 2024-06-11

**Authors:** Zhao-Da Duan, Li-Yang Zheng, Qiu-Ye Jia, Hao-Lun Chen, Dong-Yao Xu, Yu-Jia Yang, Zhi Qi, Li Yang, Chun-Yun Wu

**Affiliations:** Department of Anatomy and Histology/Embryology, Faculty of Basic Medical Sciences, Kunming Medical University, 1168 West Chunrong Road, Kunming, 650500 China

**Keywords:** Scutellarin, Neurons, Microglia, Apoptosis, JAK2/STAT3 signalling pathway, Cell biology, Neuroscience

## Abstract

Previous studies have shown that scutellarin inhibits the excessive activation of microglia, reduces neuronal apoptosis, and exerts neuroprotective effects. However, whether scutellarin regulates activated microglia-mediated neuronal apoptosis and its mechanisms remains unclear. This study aimed to investigate whether scutellarin can attenuate PC12 cell apoptosis induced by activated microglia via the JAK2/STAT3 signalling pathway. Microglia were cultured in oxygen–glucose deprivation (OGD) medium, which acted as a conditioning medium (CM) to activate PC12 cells, to investigate the expression of apoptosis and JAK2/STAT3 signalling-related proteins. We observed that PC12 cells apoptosis in CM was significantly increased, the expression and fluorescence intensity of the pro-apoptotic protein Bax and apoptosis-related protein cleaved caspase-3 were increased, and expression of the anti-apoptotic protein B-cell lymphoma-2 (Bcl-2) was decreased. Phosphorylation levels and fluorescence intensity of the JAK2/STAT3 signalling pathway-related proteins JAK2 and STAT3 decreased. After treatment with scutellarin, PC12 cells apoptosis as well as cleaved caspase-3 and Bax protein expression and fluorescence intensity decreased. The expression and fluorescence intensity of Bcl-2, phosphorylated JAK2, and STAT3 increased. AG490, a specific inhibitor of the JAK2/STAT3 signalling pathway, was used. Our findings suggest that AG490 attenuates the effects of scutellarin. Our study revealed that scutellarin inhibited OGD-activated microglia-mediated PC12 cells apoptosis which was regulated via the JAK2/STAT3 signalling pathway.

## Introduction

Ischemic stroke (IS) is an acute cerebrovascular disease in which cerebral blood vessels supply insufficient quantities of blood to the brain due to various causes, resulting in local brain tissue ischaemia, hypoxia, and necrosis. IS accounts for approximately 87% of all stroke cases. IS incidence is steadily increasing, with high mortality, morbidity, and disability rates that seriously threaten human health^1–3^. After IS, microglia (MG), which are immune cells located in the central nervous system (CNS), are the first to be activated, release a large number of inflammatory factors which cause secondary neuronal damage^4^. Over time, neuronal density within ischaemic tissue gradually decreases in response to microglial activation ^5^. Studies have shown that overactivated MG mediates persistent neuroinflammation which disturbs brain homeostasis, disrupts physiological interactions between the MG and neurones, and directly leads to neuronal apoptosis, degeneration, and dysfunction by producing neurotoxic substances; these are common pathological mechanisms of CNS-related diseases^6,7^. Therefore, identifying a neuroprotective drug that regulates and activates MG to mediate neuronal apoptosis may be an important method for reducing brain tissue damage after IS.

*Erigeron breviscapus* is one of the most commonly used Chinese herbs in Yunnan, accounting for 95% of the country’s total production^8^. Scutellarin is a flavonoid extracted from *E. breviscapus* and its structure was identified as 4,5,6-hydroxyflavone-7-glucuronide. It has many beneficial effects such as anti-oxidation, anti-inflammation, vasodilation, and anti-platelet aggregation^9^. Previous studies have demonstrated substantially this drug’s efficacy in treating cardiovascular and cerebrovascular diseases such as IS and coronary heart disease^10^. The neuroprotective effect of scutellarin on patients with brain injury caused by middle cerebral artery occlusion (MCAO) in rats has been widely studied. It can improve neurological function scores, reduce cerebral infarct volume percentage, and enhance endogenous antioxidant activity^11–13^. Another study showed that scutellarin inhibits MG-mediated neuroinflammation and reduces neuronal apoptosis in cerebral ischaemic injury, thus playing a neuroprotective role ^14,15^. However, the relevant mechanisms remain unclear. The tyrosine kinase 2 (JAK2)/signal transducer and activator of transcription 3 (STAT3) has neuron-specific functions and participates in the survival, proliferation, and differentiation of neurones under normal physiological conditions. Recent studies have shown that the JAK2/STAT3 signalling pathway is closely related to neuronal apoptosis following CNS lesions^16–18^. However, whether scutellarin can reduce neuronal apoptosis mediated by MG through JAK2/STAT3 signalling requires further study.

In conclusion, PC12 and BV-2 MG cell lines were cultured in vitro, and a BV-2 MG-conditioning medium (CM) model was established to study the changes in PC12 cells apoptosis with activated MG and the addition of scutellarin. Simultaneously, since the JAK2/STAT3 signalling pathway participates in this process, it provides an experimental basis to develop improved treatment drugs for patients with IS.

## Materials and methods

### Reagents and instruments

Scutellarin (#THT033, Ronghe Medical Technology, Shanghai, China), F12/DMEM 1:1 high sugar medium (#C3130-0500, BI, Shanghai, China ), sugar-free culture medium (#C3122-500, BI, Shanghai, China), cell culture level anhydrous glucose (#G8150, Solarbio, Beijing, China), TUNEL kit (#PF00006, Proteintech, Wuhan, China), CCK-8 cell viability assay kit (#PF00004, Proteintech, Wuhan, China), rabbit cleaved caspase-3 antibody (#9664, Cell Signaling Technology, Massachusetts, USA), rabbit anti-caspase-3 antibody (#66470-2-Ig, Proteintech, Wuhan, China), rabbit anti-Bax antibody (#WL01637, Wanle, Shenyang, China), rabbit anti-B-cell lymphoma-2 (Bcl-2) antibody (#WL01556, Wanlei, Shenyang, China), rabbit anti-JAK2 antibody (#3230S, Cell Signaling Technology, Massachusetts, USA), rabbit anti-p-JAK2 antibody (#ab32101, Abcam, Cambridge, UK), rabbit anti-STAT3 antibody (#12640, Cell Signaling Technology, Massachusetts, USA), Rabbit p-STAT3 antibody (#9145S, Cell Signaling Technology, Massachusetts, USA), murine anti-β-actin antibody (#66009-1-Ig, Proteintech, Wuhan, China), murine anti-NeuN antibody (#66,836–1-Ig, Proteintech, Wuhan, China), horseradish peroxidase (HPR)-labelled anti-rabbit IgG antibody (#SA00001-2, Proteintech, Wuhan, China), HPR-labelled anti-mouse IgG antibody (#SA00001-1, Proteintech, Wuhan, China), DAPI-containing fluorescent coated tablets (#S2110, Solarbio, Beijing, China), Cy3-labelled goat anti-rabbit IgG antibody (#SA00009-2, Proteintech, Wuhan, China), AF488-labelled goat anti-mouse IgG antibody (#SA00013-1, Proteintech, Wuhan, China). A cell incubator (#51033564, Thermo Fisher Scientific, Waltham, USA), hypoxia chamber (#27310, STEMCELL, Columbia, USA), vertical electrophoresis apparatus (#1658033, Bio-Rad, California, USA), semi-dry rotary membrane apparatus (#1703940, Bio-Rad, California, USA), imaging apparatus (#1708280, Bio-Rad, California, USA), and an inverted fluorescence microscope (#LSM 880, Zeiss, Jena, Germany).

### Cell culture

BV-2 microglia and PC12 cells were obtained from the National University of Singapore by Prof. Ling Eng⁃Ang. Frozen tubes of BV-2 MG and PC12 cells were removed from an ultra-low temperature freezer (− 80 ℃) and rapidly thawed in a 37 ℃-water bath. The cell suspension in the frozen tubes was drawn into 15 mL centrifuge tubes containing DMEM supplemented with 10% foetal bovine serum (FBS) and 1% penicillin/streptomycin, mixed, and centrifuged at 1000 rpm for 5 min. The supernatant was discarded and re-added the medium, mixed thoroughly, transferred to a cell culture flask, shaken, and incubated at 37 ℃, 5% carbon dioxide. The following day, cell survival and adherence were observed. The medium was changed and the cells were passaged at least three times before use in subsequent experiments.

### Cell viability assay

BV-2 cells were seeded at a density of 1 × 10^3^ cells/well in 96-well plates. When the cells reached approximately 90% confluence, the residual medium was washed with PBS and added to DMEM. Then, 90 μL of medium and 10 μL of CCK-8 solution were added to each well, with a well containing scutellarin, CCK-8 solution, and medium without cells was set-up as a control. Therefore, the intervention concentration of scutellarin was 0.54 μM and the duration of action was 1 h. Each group involved three duplicate wells and was incubated for 2 h. Optical density at 450 nm was measured using a microplate reader.

### Conditioned medium preparation and grouping

Routinely cultured BV-2 were seeded into 6-well plates. When the cells reached approximately 90% confluence, the BV-2 MG culture medium was replaced with DMEM without FBS. BV-2 cells were cultured in DMEM for 3 h (control group), BV-2 cells were treated with oxygen–glucose deprivation (OGD) and glucose-free medium for 2 h (CM group), and BV-2 cells were pre-protected with an optimal concentration of scutellarin for 1 h, then treated with OGD for 2 h (CM + S group) according to the CCK-8 assay. Three types of CM were worked well.

PC12 cells were routinely cultured and seeded into six- or 24-well plates. According to the CM added, PC12 cells were divided into a control group (Con), CM group (CM), and a CM + scutellarin group (CM + S). Part of PC12 cells before joining the CM, JAK2 the STAT3 signal inhibitors AG490 were added for 1 h. PC12 cells were divided into six groups: control (Con), AG490 (10 mmol/L), CM, CM + AG490, CM + scutellarin (CM + S), and CM + S + AG490. After treatment, PC12 cells were collected for protein extraction or immunofluorescence detection.

### TUNEL assay

The cells were fixed with 4% paraformaldehyde for 30 min, then 0.2% Triton PBST solution of X-100 was placed at 37 ℃ for 20 min, and TUNEL reactants were added for 2 h after 5 min, and fluorescence was observed under an inverted microscope.

### Western blot

After successful replication of the PC12 model, the cells were rinsed twice with PBS, RIPA lysate was added and cells lysed at 4 ℃ for 15 min. Cells were mechanically scraped off with a rubber scraper and ultracentrifuged at 14,000 rpm for 15 min at − 4 ℃. Protein concentrations were determined using the BCA assay. 30 μg of PC12 cell culture supernatant proteins was added to gel loading buffer, mixed well, and heated at 95℃ for 5 min. Samples were separated by 10 or 12% sodium dodecyl sulphate–polyacrylamide gel electrophoresis. Protein bands were electroblotted onto polyvinylidene difluoride membranes, blocked in 5% skim milk powder at room temperature for 2 h, then incubated with rabbit anti-cleaved caspase-3 antibody (1:1000), rabbit anti-caspase-3 antibody (1:1000), rabbit anti-Bax antibody (1:1000), rabbit anti-Bcl-2 antibody (1:1000), rabbit anti-JAK2 antibody (1:1000), rabbit anti-p-JAK2 antibody (1:1000), rabbit anti-STAT3 antibody (1:1000), or rabbit anti-p-STAT3 antibody (1:1000), and rat anti-β-actin antibody (1:5000) and incubated overnight at 4℃ on a refrigerated shaking platform. The following day, the primary antibody solution was removed and the cells were rinsed with PBS three times. HRP-conjugated goat anti-rabbit antibody (1:5000) was incubated with goat anti-mouse antibody (1:5000) for 1 h at room temperature. Cells were rinsed with PBS three times, according to the supplier's instructions. Using enhanced chemiluminescence reagents, a Bio-Rad imaging system was employed. ImageJ software was used to quantify band intensities using grey-scale values.

### Immunofluorescence staining

PC12 cells were fixed on slides using 4% paraformaldehyde for 30 min, rinsed with PBS, and blocked with 10% goat serum for 1 h. The primary antibodies are as follows: mouse anti- NeuN (1:200), rabbit anti-cleaved caspase-3 (1:200), rabbit anti-caspase-3 antibody (1:200), rabbit anti-Bax (1:200), rabbit anti-Bcl-2 (1:200), rabbit anti-JAK2 (1:200), rabbit anti-p-JAK2 (1:200), rabbit anti-STAT3 (1:200), and rabbit anti-p-STAT3 (1:200) and incubated with primary antibodies overnight in a refrigerator at 4 ℃. The primary antibody was then removed and cells incubated at 37 ℃ for 1 h with Cy3-conjugated secondary (1:200) and CoraLite 488-conjugated secondary antibodies (1:200). After washing with PBS, the slides were sealed with DAPI, observed and preserved under an inverted microscope. Immunofluorescent intensity was expressed after image quantization as integral density using ImageJ software. Each set of experiments was triplicated.

### Statistical analysis

Quantified using Image Lab and ImageJ. Data are expressed as means ± standard deviation. One-way ANOVA was used to evaluate statistical significance. GraphPad Prism 6.0 software was used for analysis.

## Results

### CCK-8 assays were used to identify the optimal scutellarin concentration for intervention

The viability of BV-2 MG cells treated with 0–13.5 μM scutellarin was determined using CCK-8 assays. No changes were noted in cell vitality between the 0.02–0.54 and 0 μM groups. Compared with the 0 μM group, the 2.7–13.5 μM cohort exhibited a significant reduction in cell viability (*p* < 0.05).

**Figure 1:**
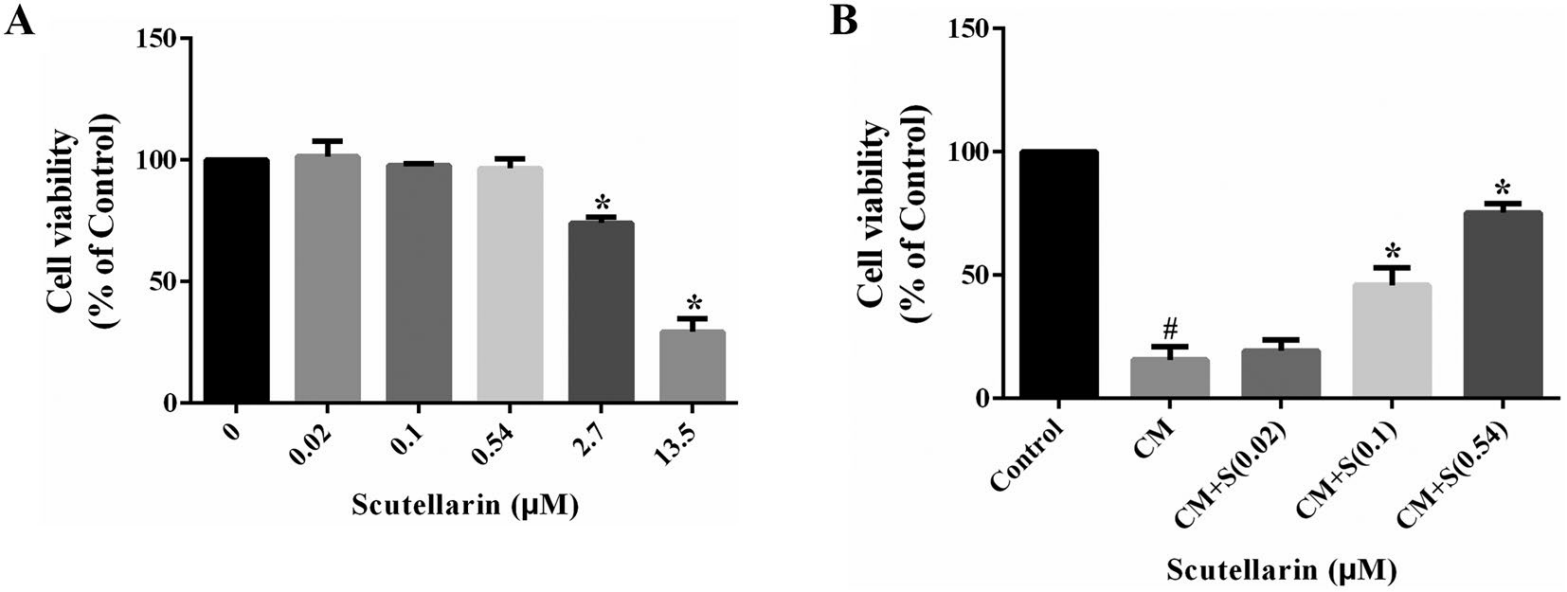
Optimum intervention concentration of scutellarin detected by CCK-8 assays. (**A**) The cell viability of BV-2 MG with different scutellarin concentrations was detected using CCK-8 assays *p < 0.05 versus (vs.) the 0 μM group. (**B**) The CCK-8 assay detects viability of PC12 cells in the CM model and the effect of scutellarin intervention #*p* < 0.05 *vs* control group; *p < 0.05 *vs* CM group.

CCK-8 assays were used to detect the viability of PC12 cells mediated by activated BV-2 MG and the impact of 0.02–0.54 μM of scutellarin. Cell viability in the CM group was significantly lower than that in the control group (*p* < 0.05). There was no significant difference in cell viability between the CM + S (0.02 μM) and CM groups. Compared with the CM group, cell viability of the CM + S (0.1 μM) and CM + S (0.54 μM) groups were significantly increased (*p* < 0.05) (Fig. 1A,B). Therefore, 0.54 μM was selected as the optimum scutellarin concentration for subsequent experiments.

### Effect of scutellarin on the expression of inflammatory factors in PC12 cells in the CM model

**Figure 2:**
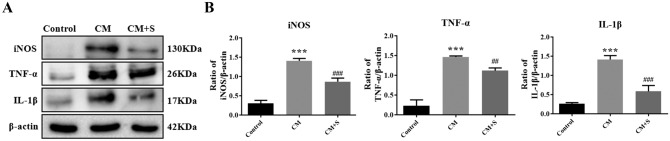
Expression changes of inflammatory factors in the CM of PC12 cells detected by western blotting. (**A**) Bands of western blotting. (**B**) Bar chart showing protein expression quantification. Data are shown as means ± standard deviation, n = 3 per group. ***p < 0.05 compared with the control group, ##*p* < 0.01, ###*p* < 0.001 compared with the CM group.

Western blotting was used to detect changes in the expression of inducible nitric oxide synthase (iNOS), TNF-α, and IL-1β in PC12 cells mediated by scutellarin and activated BV-2 MG. The results showed that the expression of iNOS, TNF-α, and IL-1β in the CM group was significantly increased (*p* < 0.05). After scutellarin intervention, the expression of iNOS, TNF-α, and IL-1β was significantly decreased (*p* < 0.05) (Fig. 2A,B). These results suggest that scutellarin significantly inhibited the activation of neuroinflammatory factors in PC12 cells mediated by activated BV-2.

### Effect of scutellarin on PC12 cells apoptosis in the CM model

**Figure 3:**
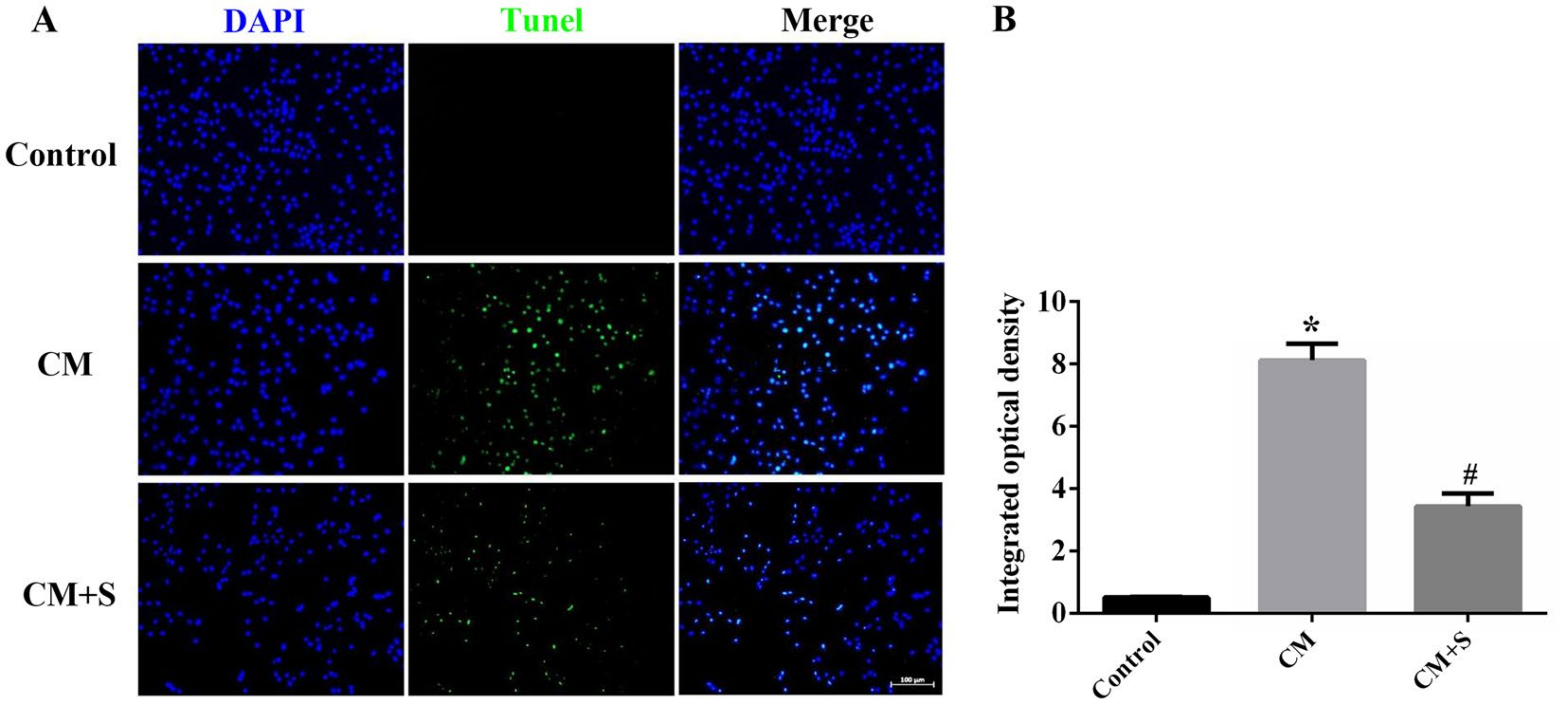
Apoptosis levels of PC12 cells detected by TUNEL. (**A**) Images of TUNEL results. Scale bar: 50 µm. (**B**) Quantitative analysis of apoptosis data for each group. Data are shown as means ± standard deviation, n = 3 per group. *p < 0.05 compared with the control group, #*p* < 0.05 compared with the CM group.

The TUNEL assay was used to detect the effect of scutellarin on PC12 cells apoptosis-mediated by activated BV-2. Our findings showed that the fluorescence intensity of TUNEL-positive cells (green fluorescence) in the CM group was significantly increased (*p* < 0.05). After scutellarin treatment, the fluorescence intensity of TUNEL-positive cells was significantly decreased (*p* < 0.05) (Fig. 3A,B), which suggests that the CM increases PC12 cell apoptosis. Apoptosis in PC12 cells was attenuated after scutellarin treatment.

### Effect of scutellarin on the expression of apoptosis-related proteins in PC12 cells in the CM model

**Figure 4:**
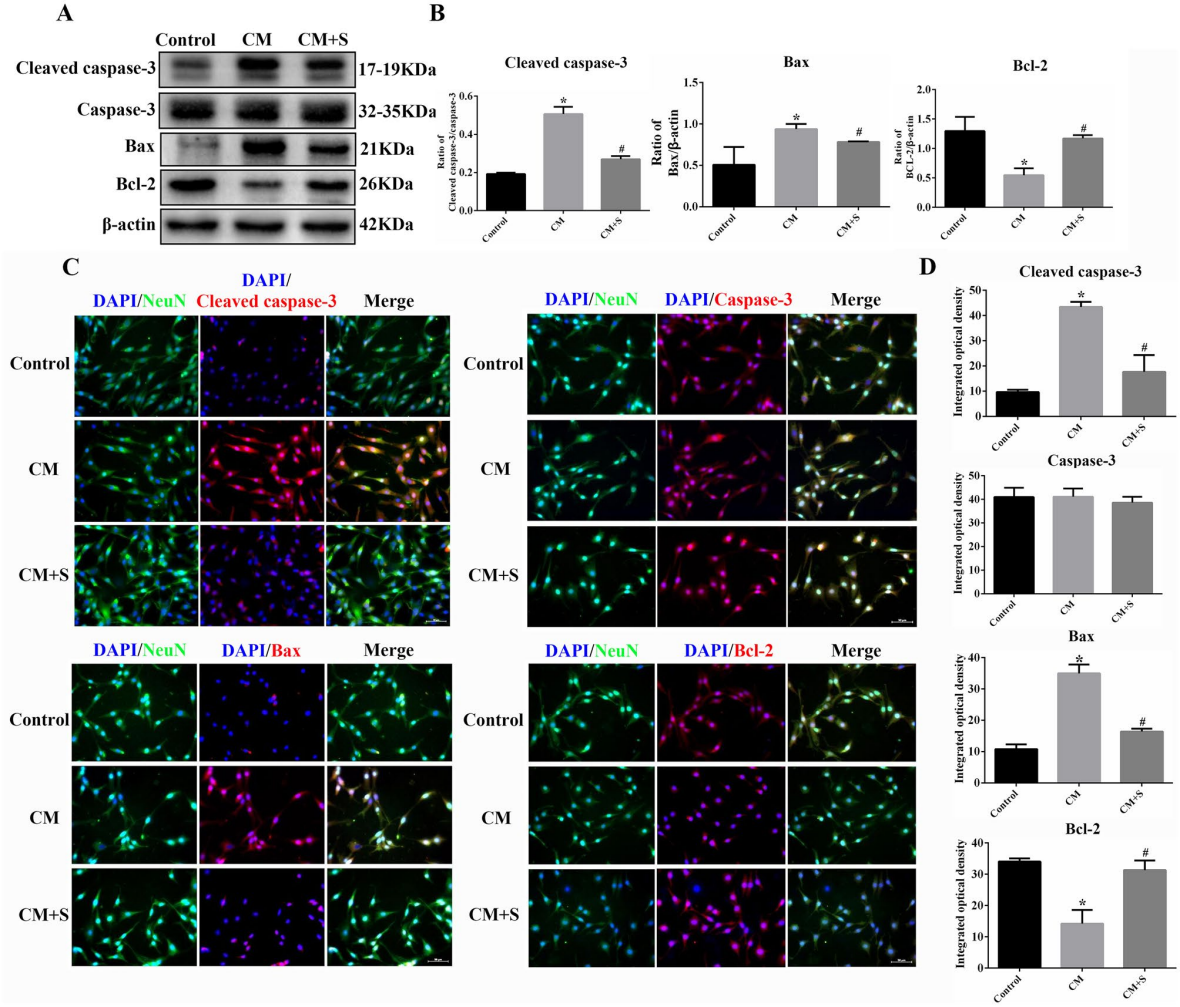
PC12 cell apoptosis related protein expression changes. (**A**) Bands of western blotting. (**B**) Bar chart of protein expression. (**C**) Images of immunofluorescent staining. Scale bar: 50 µm. (**D**) Bars of fluorescence intensity quantification. Data are shown as means ± standard deviation, n = 3 per group. *p < 0.05 compared with the control group, #*p* < 0.05 compared with the CM group.

Western blotting and immunofluorescent staining were used to detect changes in the expression of apoptosis-related proteins, including cleaved caspase-3, caspase-3, Bax, and Bcl-2. Thus, compared with the control group, the expression and fluorescence intensity of cleaved caspase-3 and Bax, in the CM group, were significantly increased, whereas Bcl-2’s expression and fluorescence intensity was significantly decreased (*p* < 0.05). Compared to the CM group, the expression and fluorescence intensity of cleaved caspase-3 and Bax were significantly decreased, whereas Bcl-2’s expression and fluorescence intensity was significantly increased post scutellarin treatment (*p* < 0.05). There was no significant difference in caspase-3 protein expression or fluorescence intensity among the groups (*p* > 0.05) (Fig. 4A–D). These results suggest that scutellarin inhibited activated BV-2 MG to mediate the expression of cleaved caspase-3 and Bax and promoted the expression of Bcl-2 in PC12 cells.

### Effects of scutellarin on JAK2/STAT3 pathway-related protein expression in PC12 cells in the CM model

**Figure 5:**
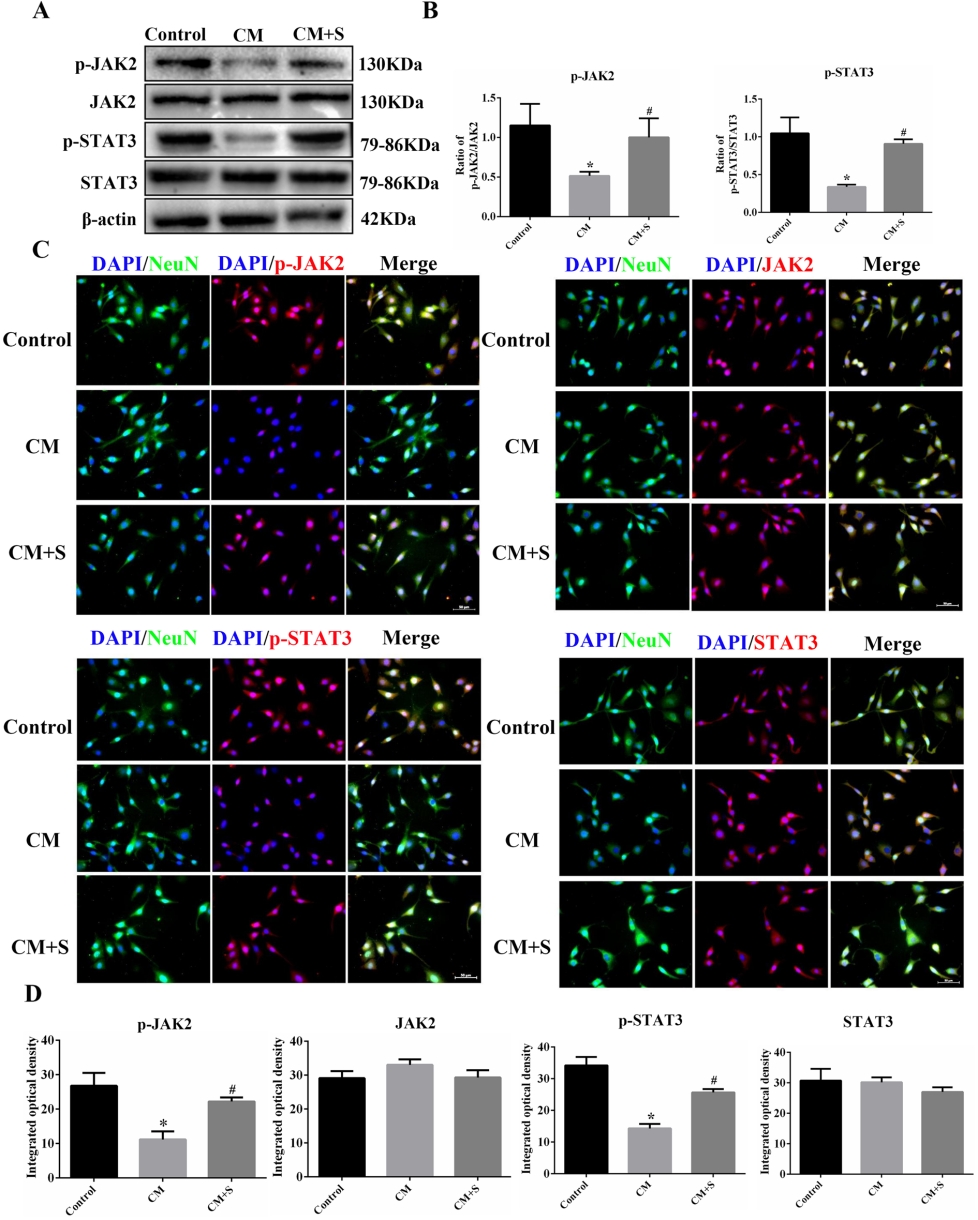
Expression changes involving JAK2/STAT3 pathway-related proteins in PC12 cells. (**A**) Bands of western blotting. (**B**) Bar chart of protein expression. (**C**) Images of immunofluorescent staining, Scale bar: 50 µm. (**D**) Bars of fluorescence intensity quantification. Data are shown as means ± standard, n = 3 per group. *p < 0.05 compared with the control group, #*p* < 0.05 compared with the CM group.

Western blotting and immunofluorescent staining were used to detect changes in the expression of JAK2/STAT3 pathway-related proteins. Thus, compared with the control group, the JAK2 and STAT3 phosphorylation levels and fluorescence intensity in the CM group were significantly decreased (*p* < 0.05). JAK2 and STAT3 phosphorylation levels and fluorescence intensity in the scutellarin intervention group were significantly higher (*p* < 0.05) than those demonstrated by the CM group. There were no significant differences in the expression and fluorescence intensity of JAK2 and STAT3 between the groups (*p* > 0.05) (Fig. 5A–D), which suggests that activated BV-2 MG inhibited the activation of JAK2 and STAT3 in PC12 cells, and that scutellarin inverted this outcome.

### Effect of AG490, on PC12 cell apoptosis

**Figure 6:**
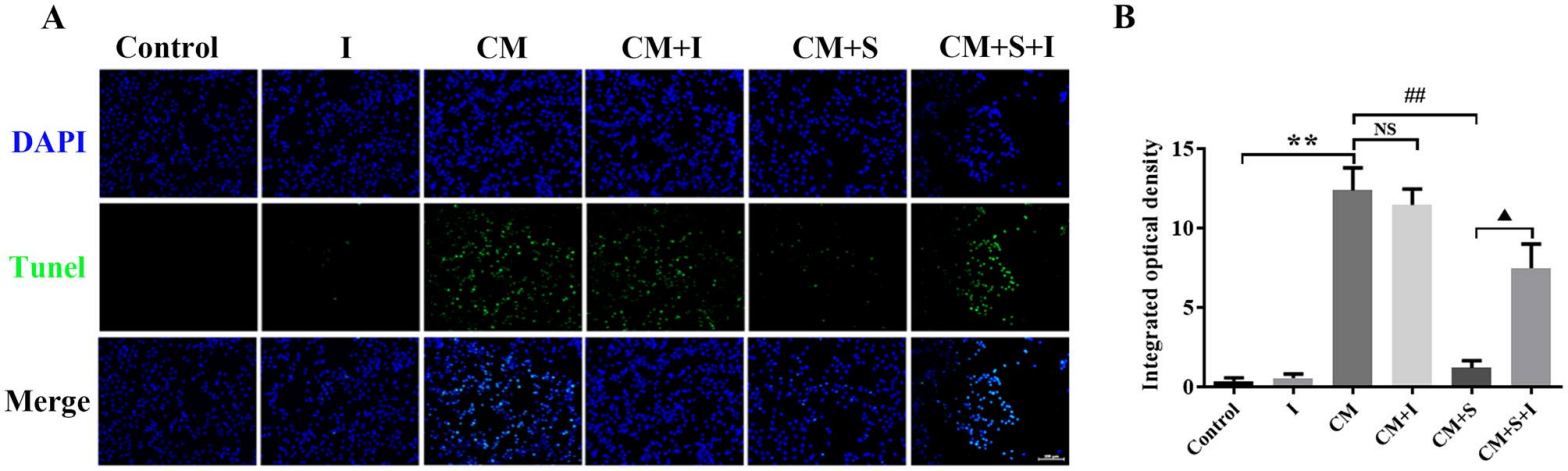
Effect of AG490 on PC12 cell apoptosis per group. (**A**) Images of TUNEL results, Scale bar: 50 µm. (**B**) Quantitative analysis of apoptosis data per group. Data are shown as means ± standard deviation, n = 3 per group. **p < 0.01 compared with the control group; ##*p* < 0.01 compared with the CM group; **NS** *p* > 0.05 compared with the CM group; **▲p** < 0.05 compared with the CM + S group.

TUNEL staining was used to detect changes in PC12 cell apoptosis. The results showed that the fluorescence intensity (green fluorescence) of TUNEL-positive cells in the CM group was significantly higher than that in the control group (*p* < 0.05). Compared with the CM group, the fluorescence intensity of TUNEL-positive cells was significantly decreased after scutellarin treatment (*p* < 0.05). Compared to the CM + S group, the fluorescence intensity of TUNEL-positive cells in the CM + S + I group was significantly enhanced post treatment with AG490 (*p* < 0.05) (Fig. 6A,B). These results suggest that AG490 promotes apoptosis in PC12 cells treated with scutellarin and attenuate scutellarin's effects.

### Effect of AG490 on the expression of JAK2/STAT3 signalling pathway-related proteins in PC12 cells

**Figure 7:**
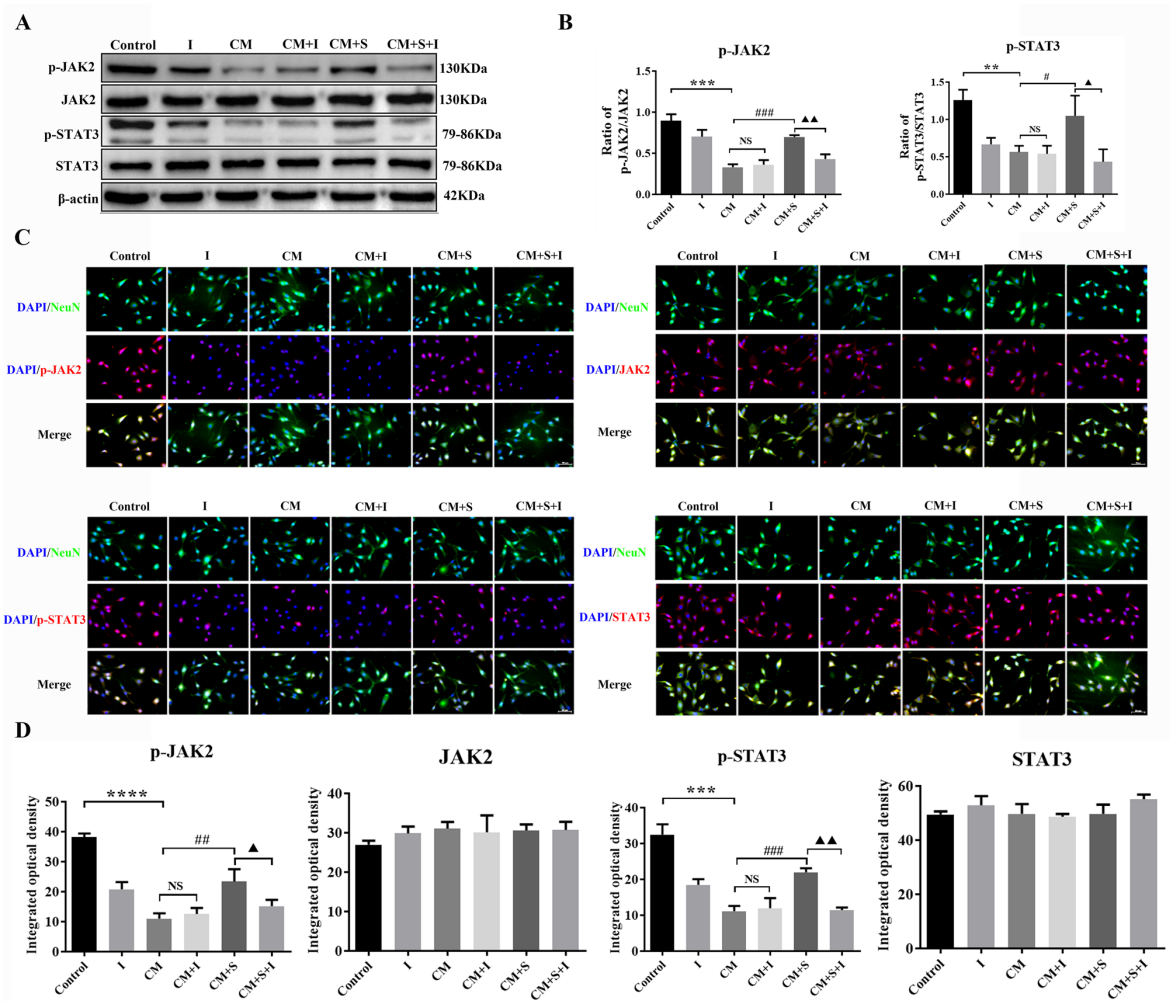
Effect of AG490 on the expression of JAK2/STAT3 signalling pathway-related proteins in PC12 cells. (**A**) Bands of western blotting. (**B**) Bar chart showing protein expression levels. (**C**) Images of immunofluorescent staining. Scale bar: 50 µm. (**D**) Bars of fluorescence intensity quantification. Data are shown as means ± standard deviation, n = 3 per group. **p < 0.01, ***p < 0.001, ****p < 0.001 compared with the control group; **##p** < 0.01, **###p** < 0.001 compared with the CM group; **NS** *p* > 0.05 compared with the CM group; **▲p** < 0.05, **▲▲p** < 0.05 compared with the CM + S group.

Western blotting and immunofluorescent staining showed that, compared with the control group, the JAK2 and STAT3 phosphorylation levels and fluorescence intensity in the CM group were significantly decreased (*p* < 0.05). Compared with the CM group, the JAK2 and STAT3 phosphorylation levels and fluorescence intensity increased significantly post scutellarin treatment (*p* < 0.05). After AG490 intervention, the JAK2 and STAT3 phosphorylation levels and fluorescence intensity were significantly lower in the CM + S + I group than in the CM + S group. The expression levels of JAK2 and STAT3 in each group were not significantly different (*p* > 0.05) (Fig. 7A–D). These results suggest that AG490 effectively inhibited activation of the JAK2/STAT3 signalling pathway in PC12 cells, thereby reducing the effects of scutellarin.

### Effect of AG490 on apoptosis-related protein expression in PC12 cells per group

**Figure 8:**
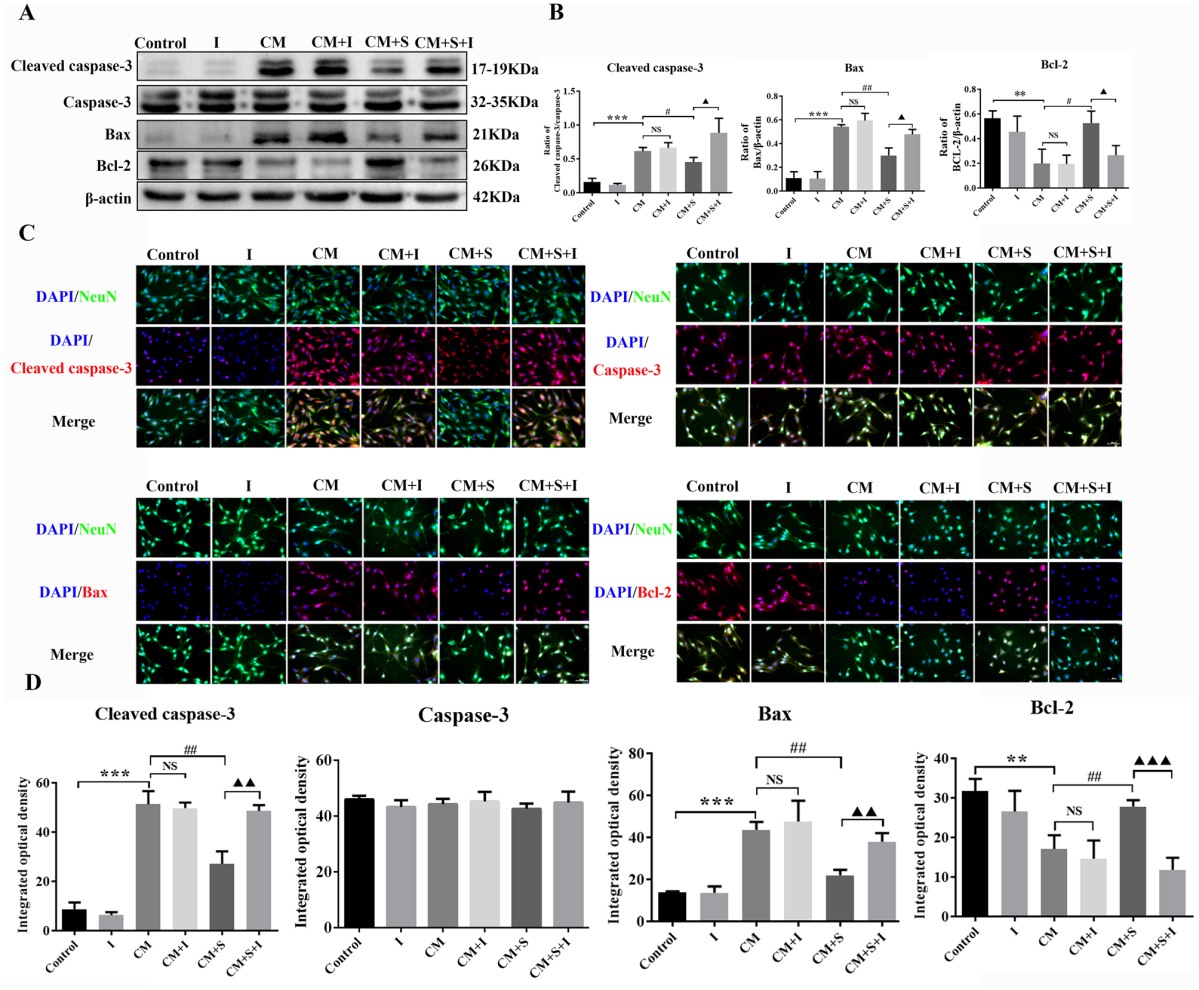
Effect of AG490 on expression of apoptosis-related proteins in PC12 cells. (**A**) Bands of western blotting. (**B**) Bar chart of protein expression. (**C**) Images of immunofluorescent staining. Scale bar: 50 µm. (**D**) Bars of fluorescence intensity quantification. Data are shown as means ± standard deviation, n = 3 per group. **p < 0.01, ***p < 0.001 compared with the control group; #*p* < 0.05, ##*p* < 0.01 compared with the CM group; **NS p** > 0.05 compared with the CM group; **▲p** < 0.05, **▲▲p** < 0.01, **▲▲▲p** < 0.001 compared with the CM + S group.

We verified that scutellarin mediates PC12 cell apoptosis via regulating activated MG through the JAK2/STAT3 signalling pathway. Western blotting and immunofluorescent staining were used to detect changes in the expression of the apoptosis-related proteins, cleaved caspase-3, caspase-3, Bax, and Bcl-2. Therefore, the expression and fluorescence intensity of cleaved caspase-3 and Bax in the CM group were significantly increased compared to the control group, while levels of Bcl-2 decreased significantly (*p* < 0.05). Compared with the CM group, the expression and fluorescence intensity of cleaved caspase-3 and Bax decreased significantly post scutellarin treatment; while Bcl-2 increased significantly (*p* < 0.05). Compared with the CM + S group, the expression and fluorescence intensity of cleaved caspase-3 and Bax in the CM + S + I group were significantly increased after AG490 intervention; while Bcl-2 was significantly decreased (*p* < 0.05). There was no significant difference in the expression of caspase-3 amongst the groups (*p* > 0.05) (Fig. 8A–D). These results suggest that scutellarin regulates activated BV-2 to mediate the JAK2/STAT3 signalling pathway in PC12 cells and regulates activated BV-2 to mediate the PC12 cell apoptosis via the JAK2/STAT3 signalling pathway.

## Discussion

After ischemic injury to the CNS, a series of pathological changes occur in the ischemic penumbra, such as excitotoxicity, excessive formation of oxygen free radicals, neuroinflammatory responses, and calcium overload, leading to neuronal apoptosis. Two aspects have attracted much attention: the apoptotic program activated by hypoxia–ischemia; the glial cells activated apoptosis of neurons. Microglia imbalances as the central nervous system injury after the first response of glial cells, the neurons apoptosis of ischemic tissues and nerve inflammation pathological process plays an important role^19–22^.

Activated MG are the predominant cause of neuronal death in the ischemic penumbra after cerebral ischemia ^23^. After a stroke, the density of neurones in the penumbra gradually decreases with MG activation ^4^. Activated MG releases large amounts of iNOS, pro-inflammatory cytokines TNF-α, IL-1β, and reactive oxygen species (ROS); amongst them, iNOS continues to produce high levels of NO ^24,25^, which inhibits cytochrome oxidase, in the mitochondria, and neuronal energy production leading to excitotoxicity and apoptosis ^25–27^. TNF-α and IL-1β are also major inflammatory proteins released by the activated MG, triggering the caspase-dependent apoptotic program through the exogenous receptor pathway in neurones ^23^. Research has found that the expression of TNF apoptosis receptor after cerebral ischaemia significantly increases; this increase was observed within 12 h after ischaemia, and within 24–48 h reached a peak, which is consistent with neuronal apoptosis^28,29^. In addition, MG expressing high levels of ROS are, after brain ischemia, the main cause of injury as they disrupted cell signalling and gene regulation, which indirectly increases damage. Additionally, they can interact with other free radicals, causing further tissue damage, and are considered to occur post the occurrence of IS trigger factors that promote inflammatory reactions and neuronal apoptosis^30^. Therefore, the inhibition of neuronal death via activated MG, is an important strategy for treating patients with stroke patients.

JAK2 is a member of the receptor-associated cytoplasmic protein tyrosine kinase family and is ubiquitous in cells. The tyrosine motif in the cytoplasmic domain of the activated JAK2 receptor is phosphorylated, creating a docking site for the Src homology 2 domain of STAT3. Followed by, the phosphorylation of STAT3 pathway proteins, which dimerise in the nucleus. It also regulates the transcription of target genes^31^. The JAK2/STAT3 signalling pathway is closely related to cell proliferation, differentiation, apoptosis, oxidative stress, and inflammation. Among these, STAT3 pathway proteins promote Bcl-2 gene expression and reduce caspase-3 activation, thus inhibiting cell apoptosis^32^. Liu et al. ^33^ confirmed that diosmin inhibits neuronal apoptosis in the brains of mice with ischemia/reperfusion injury through the JAK2/STAT3 signalling pathway in a murine MCAO animal model. Li et al. ^34^ established an animal model of bilateral common carotid artery ligation in rats and confirmed that curcumin protects neurones from apoptosis in the rat brain after ischemia/reperfusion injury through the JAK2/STAT3 signalling pathway.

Our previous in vivo and in vitro experiments have shown that scutellarin can effectively inhibit various inflammatory proteins released by MG, such as TNF-α, IL-1β and iNOS^35,36^. These proteins are likely to participate in the activation of MG-mediated neuron apoptosis pathway. Therefore, our findings showed that scutellarin reduced OGD-induced MG-mediated apoptosis of PC12 cells and promoted the activation of the pathway-related proteins p-JAK2 and p-STAT3. After the use of a pathway-specific inhibitor, AG490, the expression of pro-apoptotic proteins was upregulated, and the expression of anti-apoptotic and pathway-related proteins was downregulated, which weakened the intervention effect of scutellarin. Moreover, Marı´n-Teva and Wakselman et al. determined that as certain neurones undergo programmed cell death, they send ‘find me’ and ‘eat me’ signals to attract MG, and MG promotes caspase-3 activation in neurones and triggers neuronal phagocytosis^37,38^.

In summary, this study explored the effects of activated MG on neuronal apoptosis, the impact of scutellarin on MG mediated apoptosis, and demonstrated an in vitro CM model in which scutellarin inhibited OGD-activated BV-2 MG-mediated PC12 cell apoptosis by activating the JAK2/STAT3 signalling pathway, thus playing a neuroprotective role. This study provides an experimental basis for using scutellarin in the treatment of stroke and other related CNS diseases.

### Supplementary Information

Supplementary Figures.

## Data Availability

The datasets used and/or analyzed during the current study are available from the corresponding author on reasonable request.
