# Supplementary figures and images for: Effect of scutellarin on BV-2 microglial-mediated apoptosis in PC12 cells via JAK2/STAT3 signalling pathway
Source: Sci Rep. 2024 Jun 11;14:13430. doi: 10.1038/s41598-024-64226-x (PMC11166921; doi:10.1038/s41598-024-64226-x)

**p-JAK2 (130KDa)**

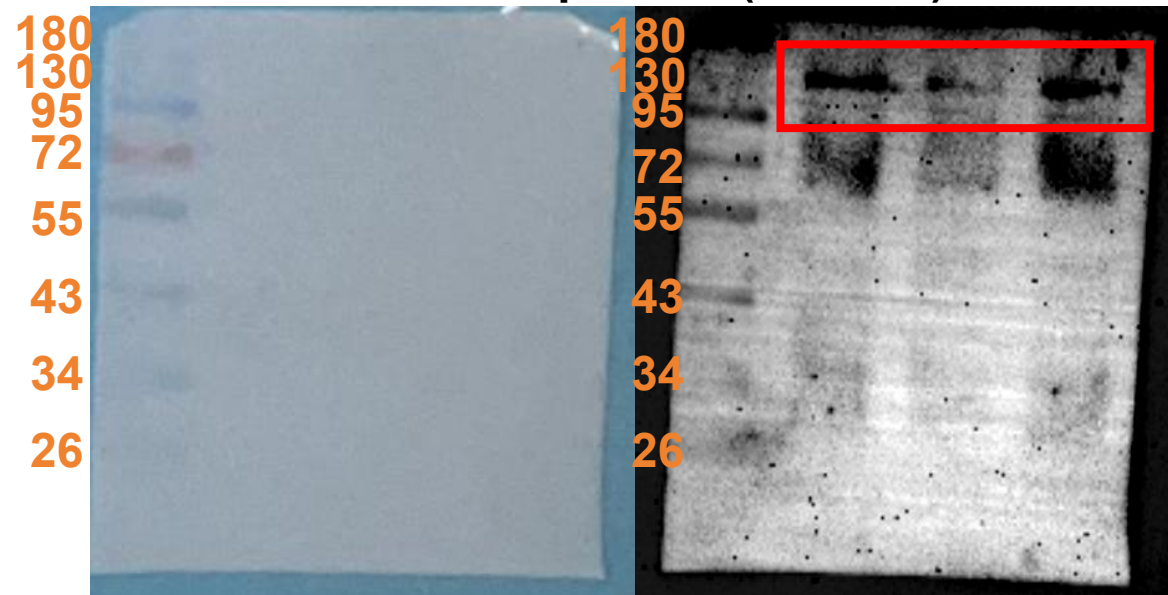

**p-STAT3 (79-86KDa)**

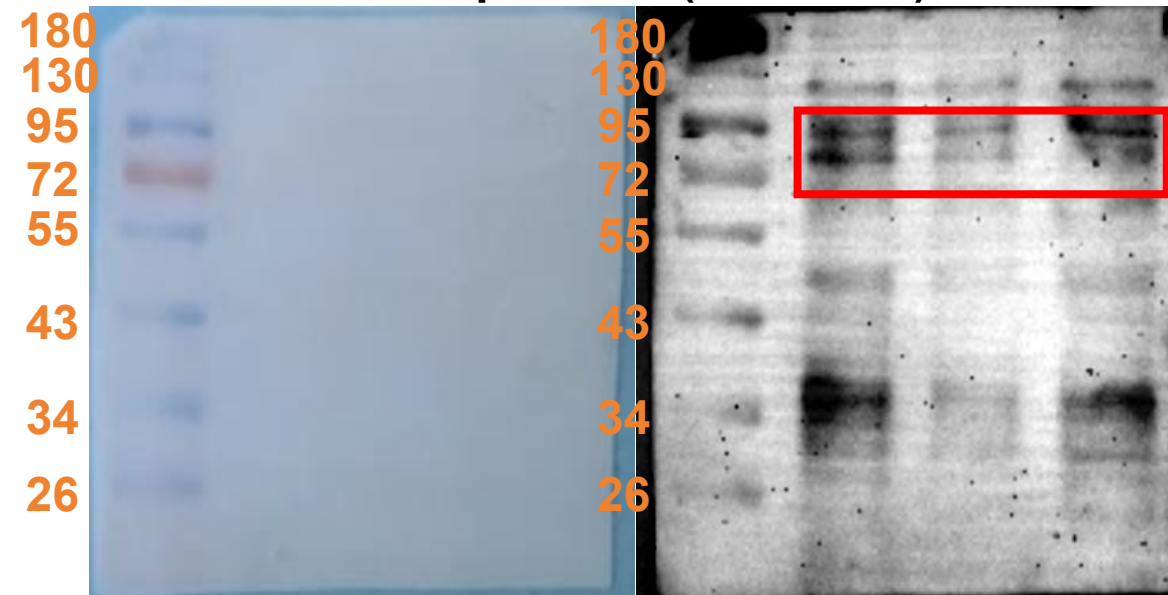

**JAK2 (130KDa)**

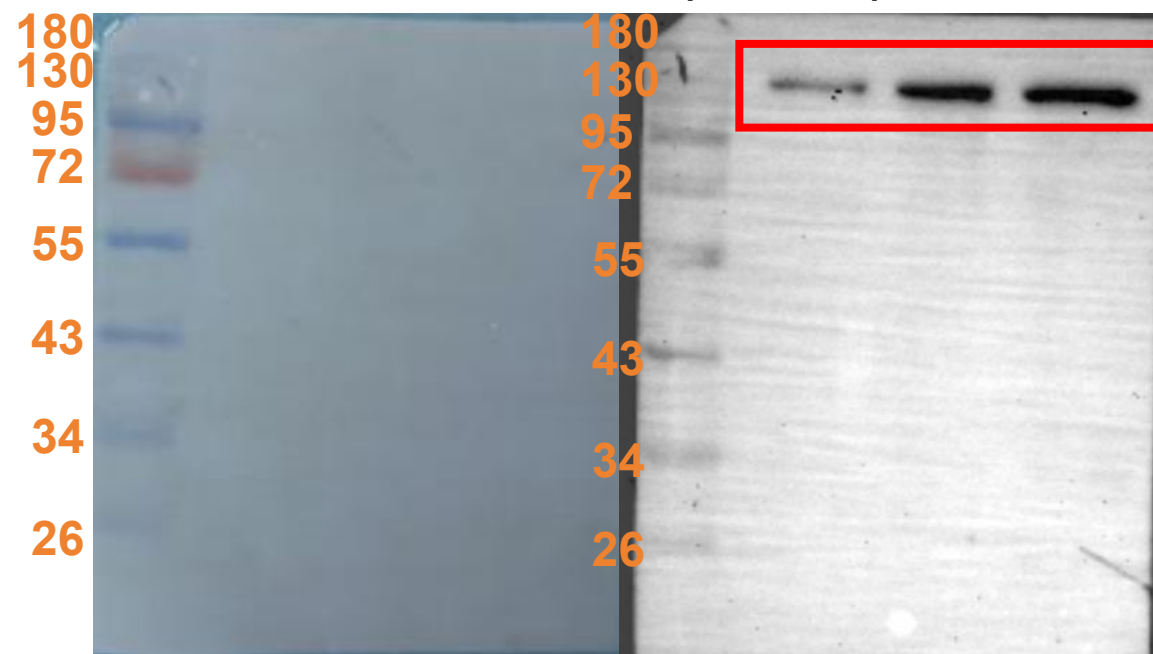

**STAT3 (79-86KDa)**

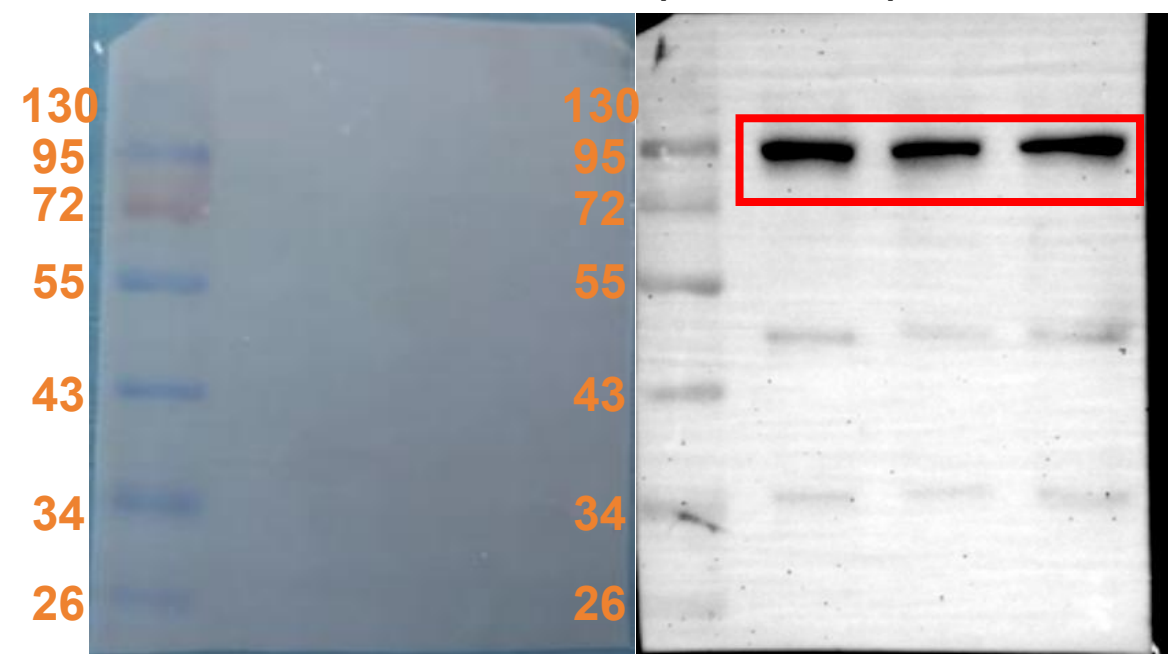

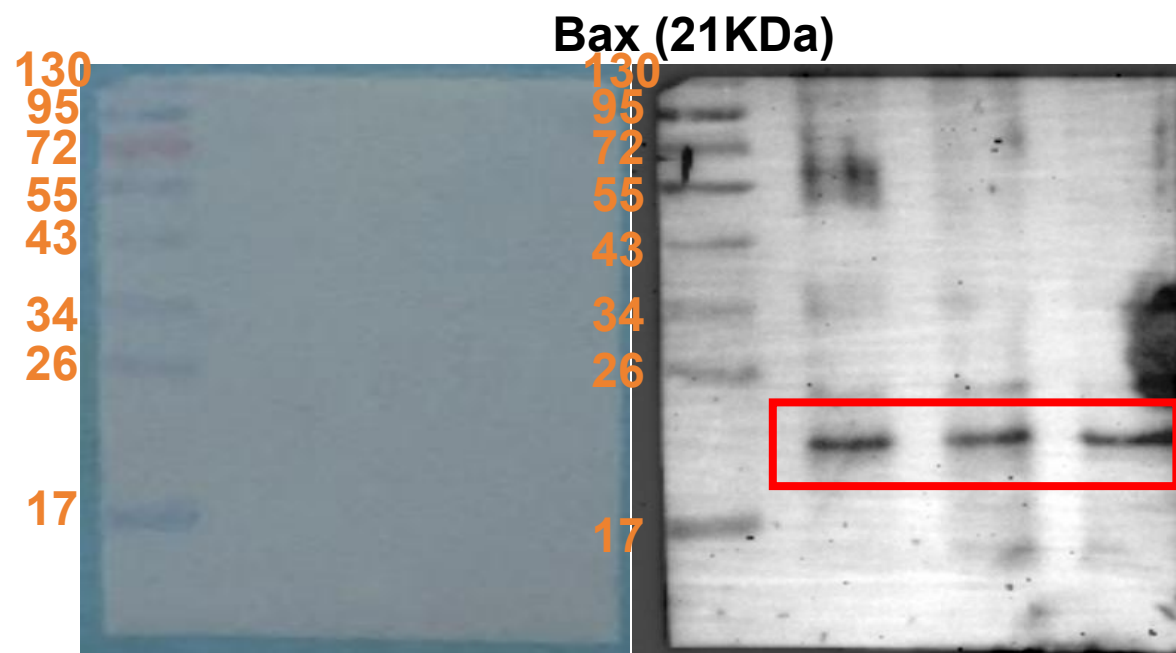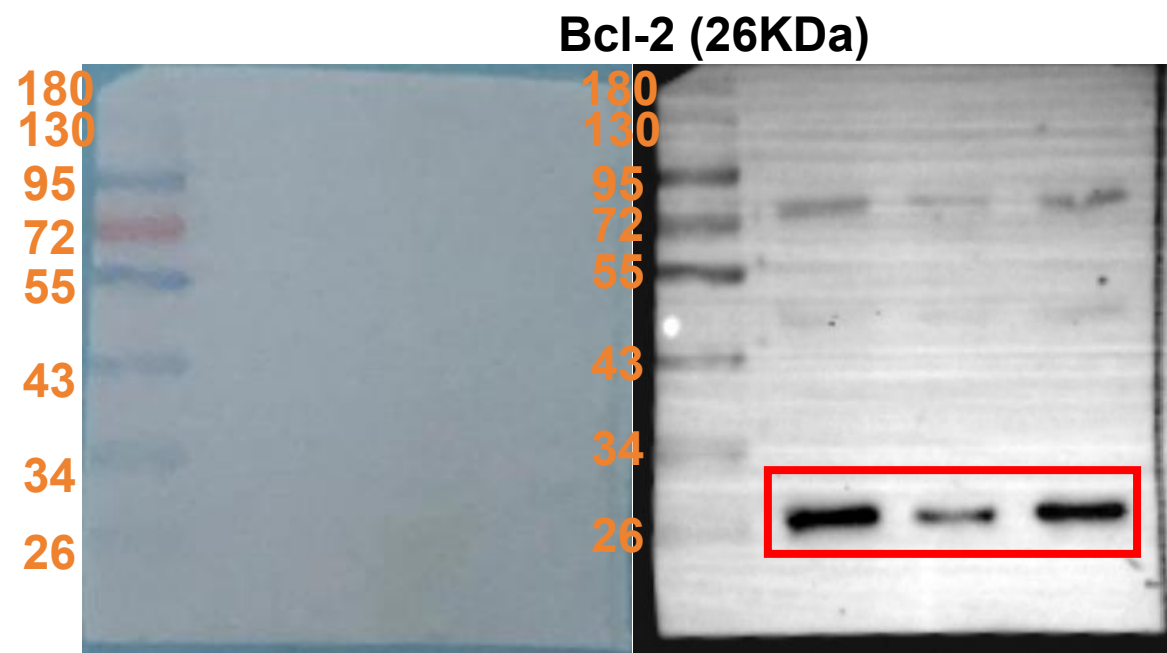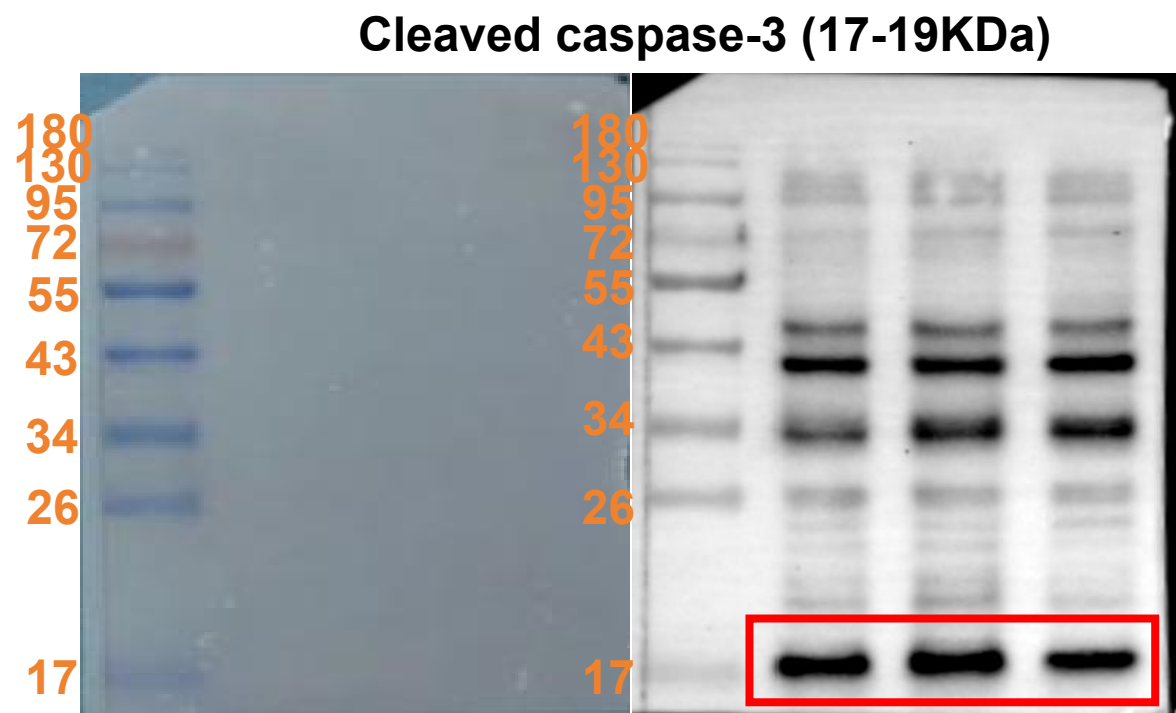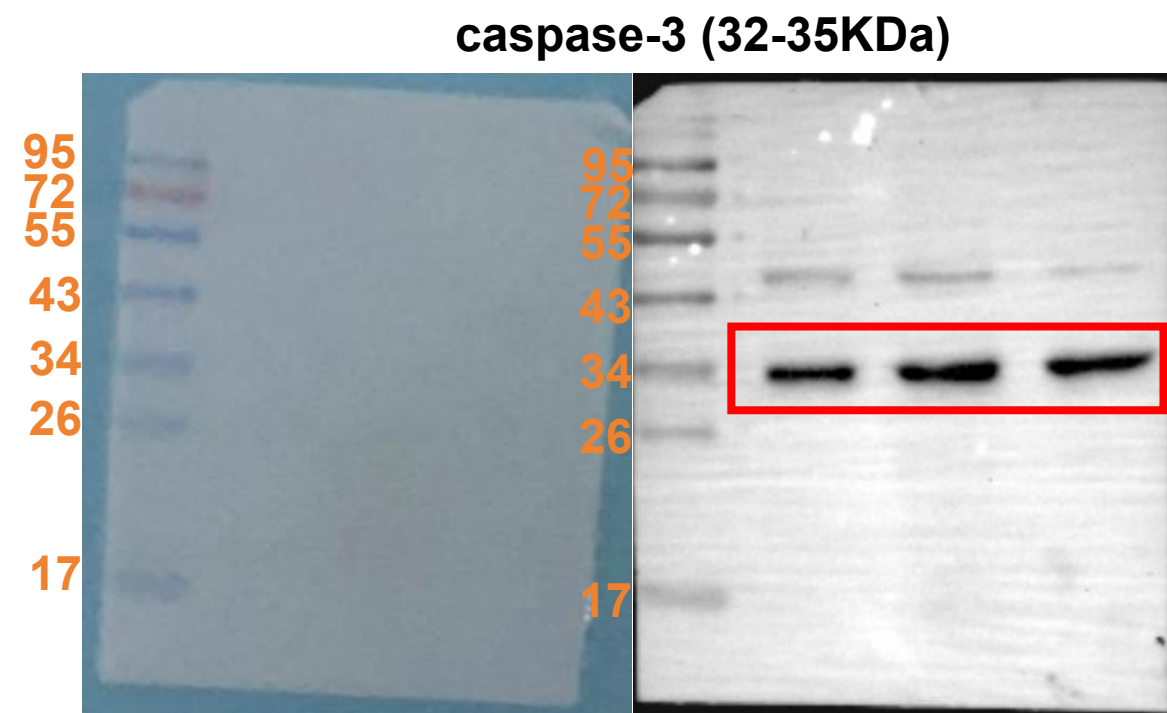

**iNOS (130KDa)**

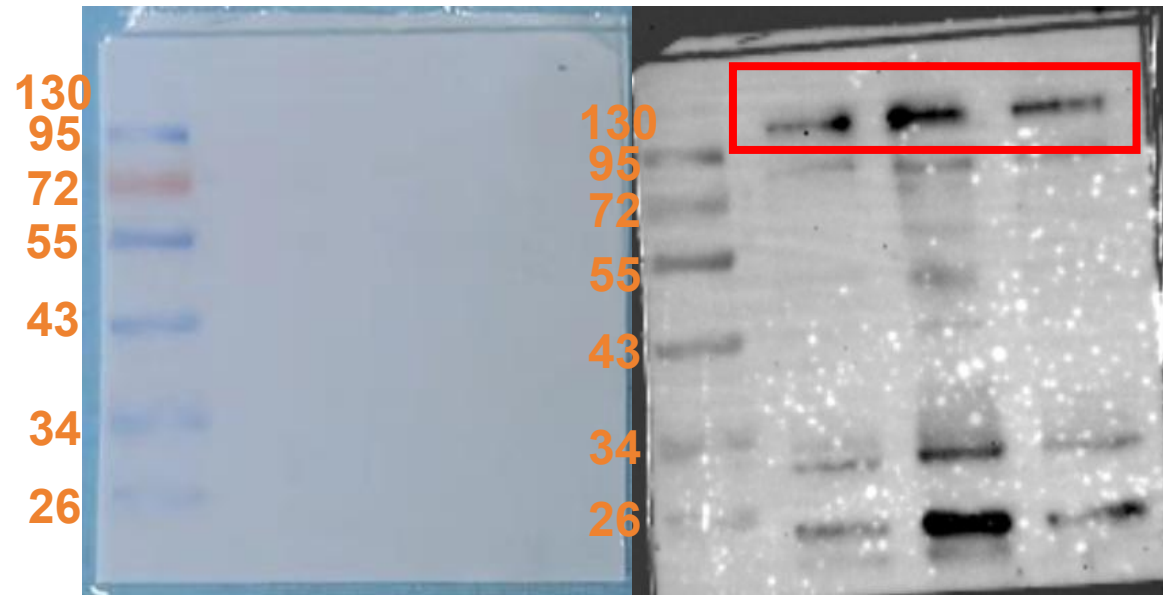

**TNF- $\alpha$  (26KDa)**

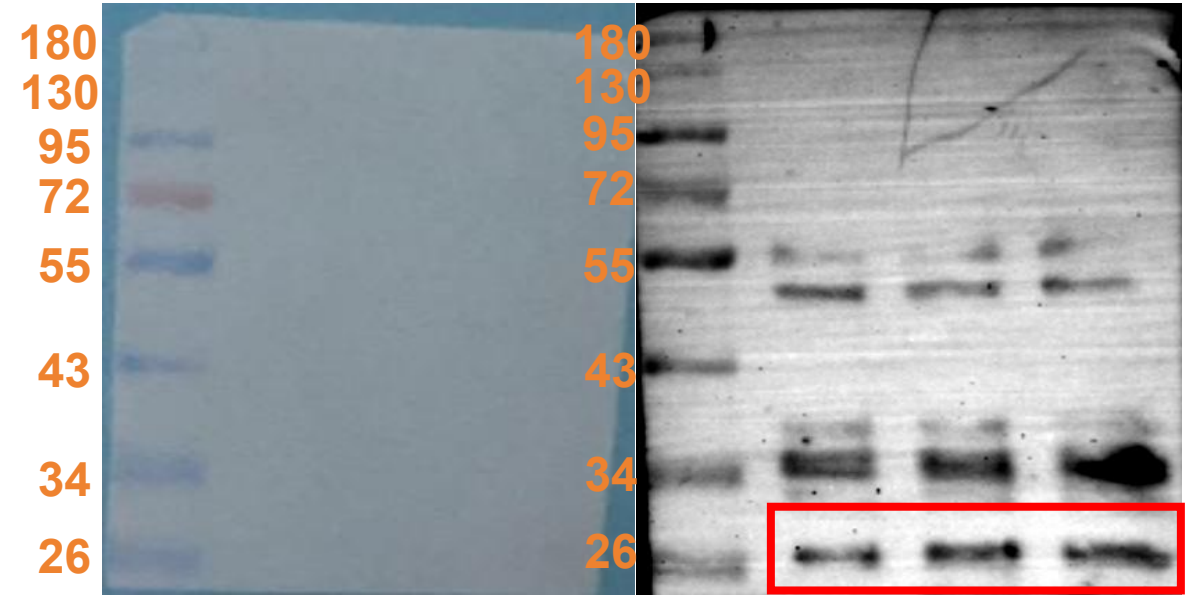

**IL-1 $\beta$  (17KDa)**

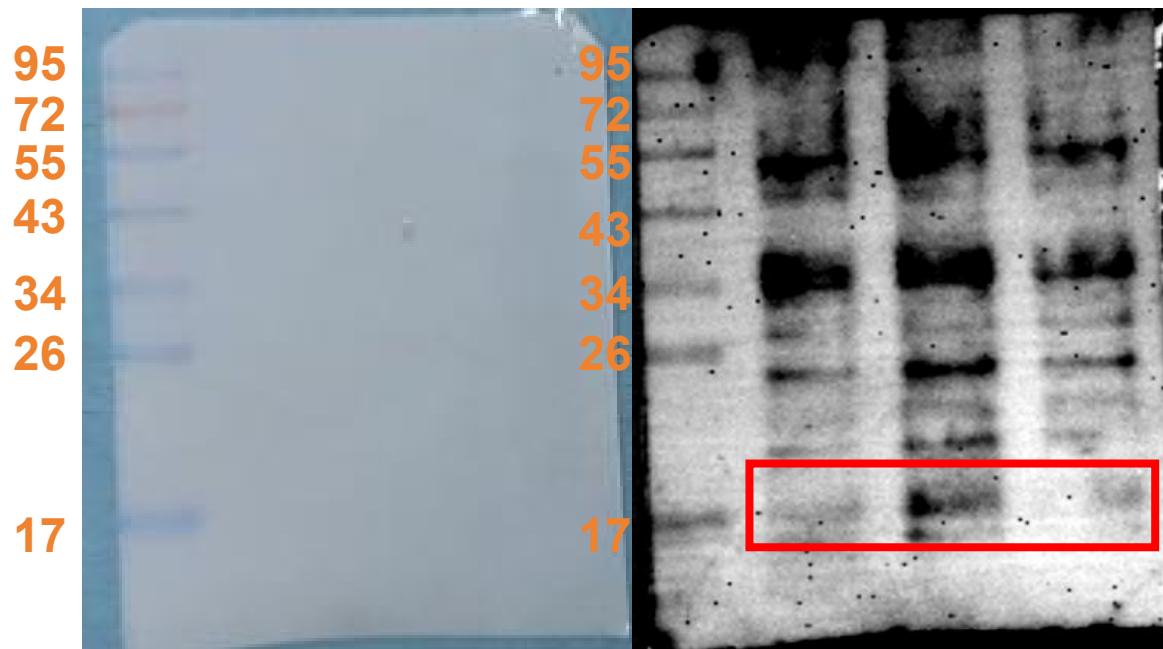

**$\beta$ -actin (42KDa)**

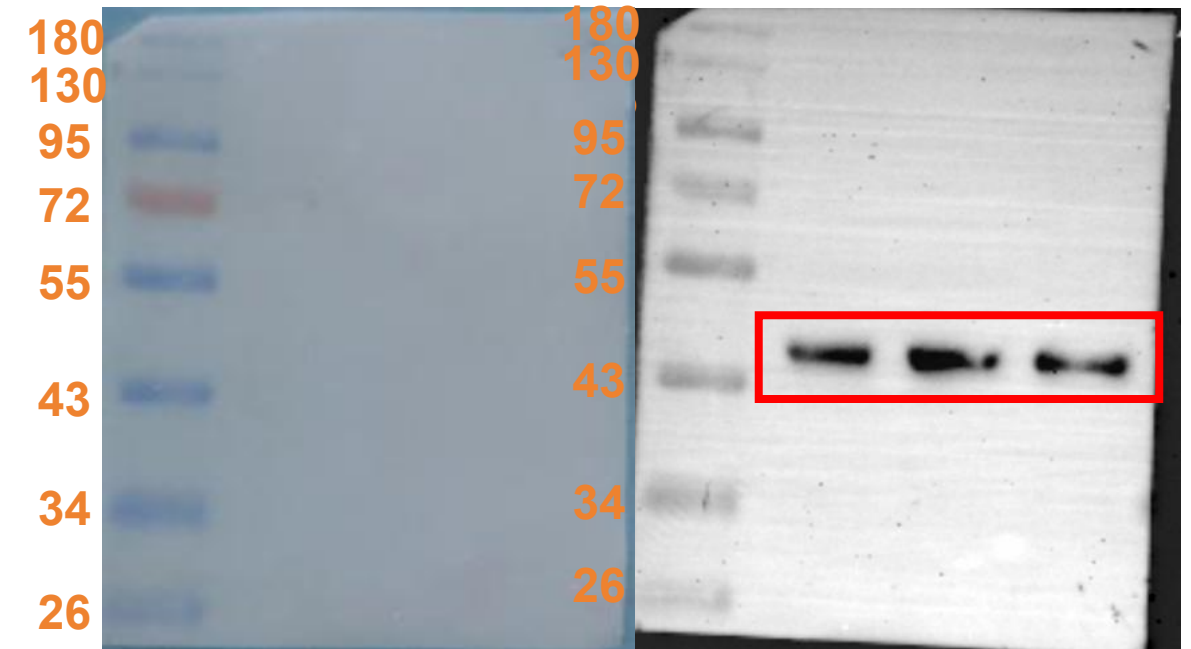

Supplement: Supplementary file 1 — Supplementary Figures. [file 41598_2024_64226_MOESM1_ESM.pdf]
